# Human bodies in virtual worlds: a systematic review of implicit sense of agency and ownership measured in immersive virtual reality environments

**DOI:** 10.3389/fnhum.2025.1553574

**Published:** 2025-08-07

**Authors:** Matteo Girondini, Marika Mariano, Giulia Stanco, Alberto Gallace, Laura Zapparoli

**Affiliations:** ^1^Department of Psychology, University of Milano-Bicocca, Milan, Italy; ^2^Mind and Behavior Technological Center, University of Milano-Bicocca, Milan, Italy; ^3^fMRI Unit, IRCCS Orthopedic Institute Galeazzi, Milan, Italy

**Keywords:** agency, ownership, virtual reality, implicit measurements, intentional binding, proprioceptive drift

## Abstract

**Introduction:**

Virtual reality (VR) offers novel tools for investigating the sense of agency (SoA) and sense of body ownership (SoO), key components of bodily self-consciousness, by enabling experimental manipulations beyond traditional paradigms. This review systematically examines how these manipulations affect SoA and SoO, focusing on their implicit indexes (e.g., intentional binding, proprioceptive drift) and their alignment with explicit measures.

**Methods:**

We clustered the manipulations based on their targeted mechanisms and evaluated their effects on SoA and SoO. Agency manipulations altered the relationship between real and virtual actions, in terms of visuomotor congruence (e.g., temporal or spatial misalignment between actions and outcomes) and movement control (e.g., replacing user actions with pre-recorded movements). Ownership manipulations focused on altering characteristics of the virtual body or limb, including physical congruence (e.g., realistic vs. object-like representations), spatial congruence (e.g., alignment of virtual and real body positions), and stimulation congruence (e.g., synchronous vs. asynchronous visuotactile feedback).

**Results:**

Agency manipulations had a strong effect on implicit SoA, while only visuomotor congruence produced a mild effect on implicit SoO. Ownership manipulations influenced implicit SoO to different extents: spatial congruence and stimulation congruence exerted moderate effects, while physical congruence showed mild effects. None of these manipulations affected implicit SoA. The alignment between implicit and explicit measures was heterogeneous, indicating that these indexes may capture distinct underlying processes. We observed that agency manipulations showed limited agreement across both SoA and SoO indexes, while ownership manipulations exhibited high agreement on SoA indexes and moderate agreement on SoO indexes.

**Discussion:**

These findings demonstrate that SoA and SoO can be functionally dissociated through targeted VR manipulations—for example, changes in body appearance did not affect implicit agency. However, SoA and SoO also show context-dependent interactions, as seen with visuomotor congruence manipulations influencing implicit SoO. This highlights their partial independence and dynamic interplay within embodied self-representation. Overall, virtual reality offers a valuable tool for exploring SoA and SoO through paradigms that overcome the limits of the traditional laboratory context. Crucially, our review identifies which types of manipulations tend to selectively influence one experience versus those that affect both, providing a framework for designing more targeted and theory-driven future studies.

## 1 Introduction

Our physical body serves as the primary bridge between our sense of self and the external world. This connection depends on neurocognitive mechanisms that ensure stability and coherence in our bodily experience over time. In everyday life, we naturally perceive our body as our own, recognizing it as the source of our voluntary actions and their effects. This bodily self-awareness relies on two dimensions: the *sense of body ownership* (SoO) and the *sense of agency* (SoA) (Gallagher, 2000). The sense of body ownership refers specifically to the feeling that one’s body is one’s own (Tsakiris, 2010). It involves a continuous and coherent experience of inhabiting a physical body, which remains unified over time despite changes in its appearance or state. In contrast, the more general sense of ownership can apply to external objects - for example, the perception that a tool or item belongs to oneself. While the general sense of ownership may occur independently of legal possession, body ownership is inherently tied to the embodied self. These concepts are not only psychologically distinct but are also underpinned by different neural mechanisms (see Turk et al., 2011, for a detailed discussion).

On the other hand, the *sense of agency* is the feeling of controlling our actions and, through them, the events in the external world (Haggard, 2017). It arises from the ability to intentionally and voluntarily initiate actions, thereby differentiating self-generated events from those caused by external forces (Moore, 2016). The dynamic interaction between SoO and SoA forms the foundation of how we experience and understand ourselves as human agents, integrating intentions, actions, and sensory feedback into a unified experience. Crucially, SoO and SoA are not fixed or static; rather, they are dynamic and context-dependent, influenced by changing sensory, cognitive, and environmental conditions. This adaptability allows us to flexibly recalibrate our sense of self in response to novel or shifting circumstances, revealing the intricate and malleable nature of how we perceive and interact with the world.

### 1.1 SoO and SoA: distinct yet interconnected dynamic processes of bodily self-awareness

Although SoO and SoA are conceptually distinct, they often co-occur and influence each other (Caspar et al., 2015). SoO arises from the integration of sensory signals, such as vision, touch, and proprioception, into a coherent bodily representation (Blanke et al., 2015). SoA, in contrast, is closely linked to volition and emerges from the temporal and spatial contingencies between actions and their outcomes (Haggard et al., 2002; Farrer et al., 2013). The body thus plays a central role in both mechanisms, mediating interactions between the self and the environment.

Crucially, both SoO and SoA are shaped by an interplay of bottom-up sensory information and top-down mechanisms (Tsakiris, 2010). Bottom-up processes depend on the timing and location of sensorimotor events, while top-down processes involve cognitive representations and prior experience. This integration dynamically updates our perception of the body and its actions.

Experimental paradigms introducing incongruences in sensory inputs or disrupting action-outcome contingencies offer compelling evidence for this flexibility. Crucially, the mismatches between expected and actual sensory information can affect SoO and SoA experiences differently. For instance, the ‘rubber hand illusion’ shows that synchronized visual and tactile input can induce SoO over a fake hand (Botvinick and Cohen, 1998; Pavani et al., 2000). Similarly, SoA can be manipulated by altering the temporal or spatial relationship between an action and its outcome (for a review, see Seghezzi et al., 2019a,b).

These findings highlight the critical role of spatiotemporal information of events occurring between the body and the external environment in shaping both SoO and SoA. The plausibility of sensory and contextual information is evaluated in relation to the brain’s internal model of the body and its dynamic interactions with the environment. This process involves sensory inputs, prior experiences, and cognitive representations used to maintain a coherent sense of self. Current research increasingly adopts experimental designs that selectively disrupt or alter these components, providing insight into the flexible and constructive nature of bodily self-awareness.

### 1.2 Virtual reality as a game-changer for studying SoO and SoA

Over the past two decades, virtual reality (VR)^1^ has revolutionized the study of SoO and SoA by leveraging embodiment procedures, which enables individuals to experience the virtual body as if it were their own. Many studies have demonstrated how VR can successfully induce ownership over both specific body parts (such as in the virtual rubber hand illusion; Sanchez-Vives et al., 2010) and entire bodies (as seen in the full body illusion; Ehrsson, 2007; Lenggenhager et al., 2007; see also the reviews by Mottelson et al. (2023), Pyasik et al. (2022)). Similarly, SoA in VR refers to the perceived control over actions performed by one’s virtual body, emerging from the spatio-temporal coherence between intentional movements and their effects in VR (Kokkinara et al., 2015).

Crucially, VR allows the creation of scenarios that go beyond the limitations of traditional experimental settings (and more in general of the physical body and environment). First, VR enables precise control over various aspects of body manipulation, involving multisensory components (visual, tactile, proprioceptive, auditory, and even nociceptive), spatial congruence of virtual body part (Antoš et al., 2024) and sensorimotor interaction features (see Gallace et al., 2011; Gallace and Spence, 2014; Girondini et al., 2024a,b). Importantly, these conditions extend beyond the constraint of classic SoO and SoA manipulations, such as the (moving) rubber hand illusion. Second, VR has the power to bend the fundamental laws of the physical world, enabling SoO and SoA explorations in conditions that would otherwise be implausible (or even impossible). For instance, VR can create scenarios where the features of the embodied avatars conflict with the individual’s actual body state or with prior knowledge about her/his own body and behavior. Such fine-grained control over experimental manipulations has opened up new possibilities for understanding how the brain adapts to discrepancies between the real and virtual body and altered body-environment interaction patterns. By inducing conflicts between the virtual body and the real body, VR allows the investigation of the brain’s adaptive mechanisms for resolving such discrepancies (Kilteni et al., 2015). This sheds light on the boundaries and limits of what can be experienced as one’s own body and under one’s control, offering critical insights into the mechanisms of SoO and SoA.

The implications of this work extend beyond basic research. VR-based paradigms provide critical insights into clinical conditions marked by disrupted self-representation, such as schizophrenia or post-stroke disorders (e.g., delusion of control, somatoparaphrenia; De Vignemont, 2011; Moore and Fletcher, 2012). They also inform research on altered states of consciousness (e.g., under psychotropic substances; Ho et al., 2020) and support the development of intuitive control systems for prosthetics and robotic avatars (Rognini and Blanke, 2016).

### 1.3 How to assess SoO and SoA in real and virtual experiments

Experimental paradigms for investigating SoO and SoA can be grouped based on the measures used to quantify these experiences. Explicit measures capture conceptual and interpretative judgments of agency and/or ownership. These typically involve direct questions that ask participants to evaluate their subjective experiences, such as the extent to which they felt a virtual limb belonged to them or how strongly they perceived themselves as the source of a specific outcome resulting from their actions (Kilteni et al., 2013). While useful, these measures rely on introspection and are sensitive to individual differences in interpretation, potentially reducing comparability across participants and conditions.

Implicit measures, by contrast, capture more automatic or pre-reflective aspects of bodily self-awareness. For SoA, one widely used implicit measure capitalize on the *intentional binding phenomenon* (i.e., the perceived compression of time between an action and its outcome, Haggard et al., 2002). For SoO, common measures include *proprioceptive drift*, in which perceived limb position shifts toward a virtual counterpart after embodiment (Tsakiris et al., 2006), and physiological responses to threat, such as skin conductance changes when a virtual body is endangered (Armel and Ramachandran, 2003). These measures offer more subtle insights but may tap into mechanisms distinct from those assessed explicitly.

Several studies suggest that explicit and implicit measures reflect different facets of ownership and agency. In SoA research, this distinction is often framed in terms of the feeling of agency (a low-level, sensorimotor-based experience) versus the judgment of agency (a higher-level attribution based on beliefs and reasoning; Synofzik et al., 2008; Moore et al., 2012; Dewey and Knoblich, 2014; Saito et al., 2015). Implicit measures such as intentional binding are typically better suited to capturing the former, while explicit self-reports more strongly reflect the latter.

A similar dissociation exists in SoO. For instance, proprioceptive drift can occur in the rubber hand illusion even when participants do not report feeling of ownership over the fake hand (Holle et al., 2011). Neuromodulation studies reinforce this divide: repetitive transcranial magnetic stimulation (rTMS) has been shown to enhance (Kammers et al., 2009) or diminish (Wold et al., 2014) proprioceptive drift toward a fake hand without altering subjective reports of ownership. A recent meta-analysis by Tosi et al. (2023) confirmed a weak correlation between questionnaire-based ownership ratings and proprioceptive drift, supporting the idea that these measures engage different underlying processes.

It is also important to distinguish between behavioral and physiological implicit measures. While both are considered “implicit,” they may reflect different dimensions of bodily self-consciousness. Behavioral measures such as intentional binding and proprioceptive drift are closely linked to sensorimotor prediction and multisensory integration, respectively. In contrast, physiological measures like skin conductance responses (SCR) capture autonomic emotional and defensive reactions, often interpreted as affective indexes of bodily self-attribution (Armel and Ramachandran, 2003; Gallace and Bellan, 2018). These responses may reflect a more pre-conscious mechanism that signals the perceived threat to a self-attributed body part.

Despite their value, implicit measures remain underutilized in VR studies on SoO and SoA. The mechanisms through which VR manipulations influence these measures are not yet fully understood, highlighting a key area for future research.

### 1.4 Aim of the review

The literature on VR paradigms for investigating SoO and SoA experiences has now reached a level of maturity that allows for systematic reviews to synthesize findings. While some reviews have already focused on VR paradigms for exploring SoO and SoA (Mottelson et al., 2023), they have generally targeted these paradigms broadly, without focusing on the innovative manipulations enabled by VR. Moreover, these reviews have only considered explicit, self-reported measures of SoO and SoA, leaving a gap in assessing the effectiveness of VR-based paradigms in modulating their implicit dimensions. This oversight is notable given extensive evidence across disciplines that explicit and implicit measures often reflect distinct underlying processes and do not always converge (Wold et al., 2014). Therefore, focusing solely on explicit outcomes may provide an incomplete picture of how VR-based paradigms engage the neurocognitive mechanisms of bodily self-awareness.

The present review aims to fill these gaps by providing a comprehensive assessment of VR’s contributions to the study of SoO and SoA, with a particular emphasis on paradigms that extend beyond the limitations of traditional laboratory setups. Specifically, we focus on VR-specific manipulations that can generate implausible scenarios—for example, by altering the physical properties of movement or creating anatomically impossible bodies. We evaluate the effectiveness of these manipulations in modulating implicit experiences of SoO and SoA and, where applicable, examine how these effects correspond to similar changes within the same construct (e.g., intentional binding and agency judgments; PD/SCR and ownership judgments). Finally, the review contextualizes these findings within established theoretical frameworks that describe the mechanisms underlying bodily agency and ownership.

Importantly, while this review adopts a systematic methodology for study selection, screening, and categorization, it does not aim to comprehensively cover all embodiment procedures in VR. Such broad overviews have been provided by recent reviews of the literature (e.g., Matamala-Gomez et al., 2019; Mottelson et al., 2023; Pyasik et al., 2022). Instead, our focus is specifically on studies that use immersive VR-based experimental manipulations designed to extend beyond traditional laboratory paradigms and that assess the SoA and SoO through implicit measures.

The decision to include only studies using immersive VR was driven by the need to ensure greater methodological consistency across the selected studies, minimizing potential variability arising from differences in experimental settings rather than from the manipulations of interest. Moreover, we only included studies in which the manipulations were exclusively enabled by immersive VR, in line with the aim of this review to complement existing broader literature on SoA and SoO in virtual environments.

## 2 Materials and methods

We interrogated the PubMed, Scopus, and Web of Science databases in May 2025. To identify as many relevant possible studies, we employed various combinations of keywords: (“agency” AND “avatar”), (“agency” AND “virtual reality”), (“intentional binding” AND “avatar”), (“intentional binding” AND “virtual reality”), (“sense of agency” AND “avatar”), (“sense of agency” AND “virtual reality”), (“sense of ownership” AND “virtual reality”), (“sense of body ownership” AND “virtual reality”), (“sense of body-ownership” AND “virtual reality”). The review was not pre-registered.

The initial full research provided 880 papers. After removing duplicates, we conducted a detailed screening of the remaining records using the Rayyan platform.^2^ Based on the title and abstract, a preliminary pool of 92 studies was retained. We then reviewed the full texts of these articles. Three independent researchers (MG, MM, and GS) screened and classified the studies, while a fourth researcher (LZ) resolved any discrepancies. During this phase, additional relevant papers were identified through a manual review of reference lists and subsequently included in the final selection.

The screening process was independently conducted by the authors based on the following inclusion criteria:

• Presence of experimental manipulations targeting the sense of agency and/or the sense of body ownership applied within an immersive VR environment.
• Presence of manipulations uniquely enabled by immersive VR, meaning they could not be replicated within traditional laboratory settings.
• Presence of primary outcome measures specifically assessing SoA and/or SoO.
• Presence of implicit measures used to assess SoA or SoO experiences (e.g., intentional binding, proprioceptive drift, or skin conductance responses).

The following exclusion criteria were applied:

• Absence of implicit measures, with assessment of SoA and/or SoO relying solely on self-report questionnaires.
• Presence of mixed or augmented reality environments, rather than fully immersive virtual reality.
• Presence of manipulations that could be implemented in traditional laboratory settings, lacking the unique affordances of immersive VR.
• Presence of samples consisting exclusively of children or clinical populations, without the inclusion of healthy adults.

**FIGURE 1:**
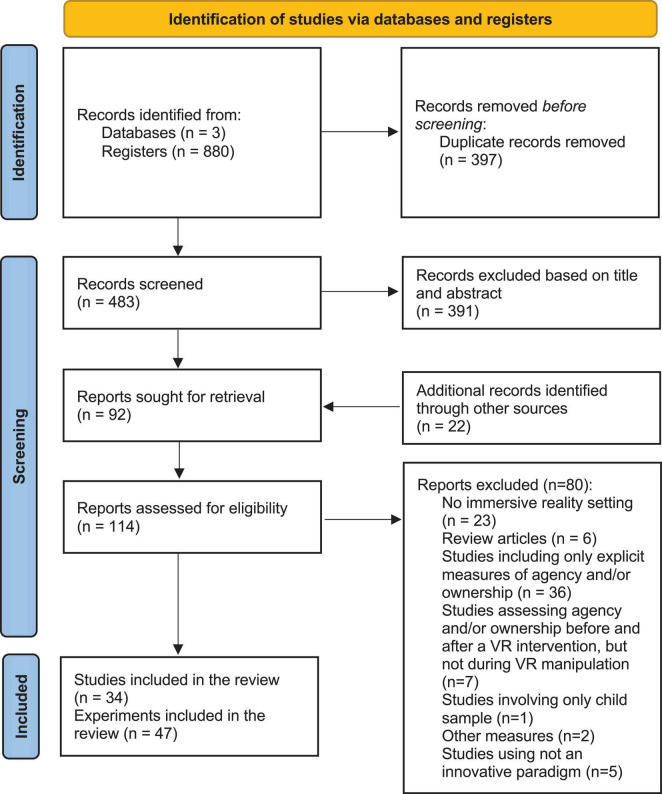
PRISMA flow diagram, summarizing the procedure followed for selecting the studies that were included in the review.

The final dataset included 34 papers (see Table 1). For details on the selection process and screening of studies, see the PRISMA diagram (Figure 1).

**TABLE 1:** Summary of reviewed experiments, dependent variables (Sense of Agency-SoA and Sense of Body Ownership- SoO), outcome measures, presence or absence of agency and ownership manipulations, and the type of VR technology employed.

	First author (year)	Dependent variable		Output measure		Agency manipulations	Ownership manipulations			Technology employed
		SoO	SoA	Implicit mesaure	Explicit meausure	Real body/Virtual body action-outcome congruence	Real body/Virtual body physical congruence	Real body/Virtual body spatial congruence	Real body/Virtual body stimulation congruence	
1	Debarba et al. (2017)	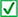	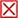	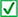	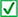	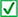	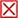	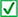	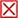	Head-mounted displays (Oculus DK2)
2	Ehrsson (2007) - Exp2	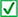	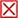	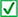	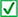	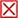	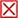	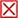	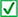	Head-mounted displays
3	Frisco et al. (2024)	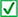	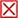	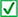	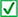	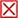	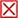	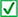	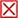	Head-mounted displays (Oculus Quest 2)
4	Grechuta et al. (2019)	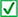	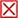	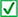	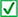	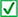	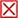	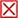	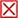	Head-mounted displays (HTC Vive)
5	Guterstam and Ehrsson (2012) - Exp3a	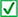	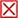	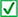	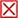	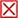	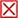	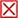	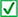	Head-mounted displays
6	Guterstam and Ehrsson (2012) - Exp3b	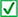	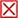	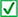	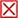	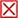	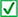	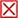	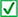	Head-mounted displays
7	Hapuarachchi et al. (2023)	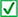	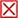	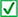	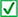	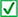	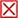	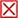	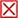	Head-mounted displays (HTC Vive Pro Eye)
8	Hara et al. (2015) - Exp2	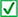	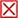	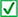	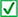	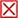	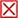	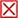	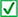	Head-mounted displays (HMZ-T1)
9	Hara et al. (2015) - Exp3	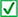	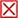	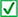	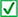	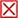	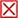	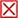	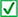	Head-mounted displays (HMZ-T1)
10	Hartfill et al. (2024)	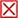	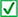	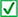	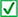	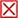	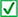	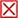	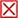	Head-mounted displays (Oculus Quest 2)
11	Heydrich et al. (2013) - Exp2	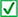	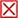	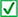	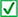	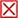	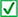	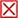	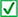	Head-mounted displays (Virtual Viewer 3D)
12	Kilteni et al. (2012)	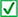	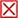	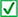	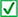	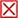	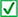	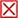	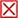	Head-mounted displays (NVIS nVisor SX111)
13	Kondo et al. (2018) - Exp3	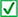	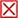	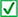	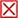	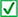	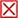	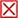	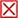	Head-mounted displays (Oculus Rift DK2)
14	Kondo et al. (2020a) - Exp1	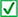	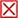	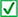	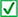	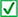	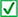	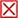	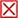	Head-mounted displays (HTC Vive Pro)
15	Kondo et al. (2020a) - Exp2	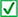	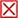	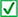	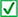	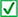	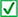	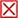	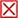	Head-mounted displays (HTC Vive Pro)
16	Kondo et al. (2020a) - Exp3	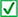	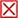	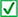	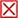	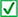	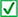	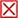	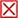	Head-mounted displays (HTC Vive Pro)
17	Kondo et al. (2020b)	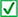	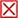	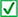	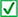	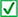	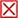	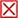	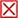	HMD Oculus Rift DK2
18	Lenggenhager et al. (2007) - Exp1	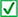	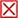	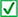	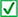	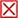	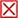	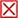	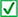	Head-mounted displays; i-glasses Video 3D Pro
19	Lenggenhager et al. (2007) - Exp2	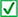	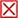	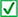	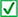	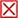	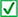	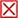	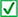	Head-mounted displays; i-glasses Video 3D Pro
20	Ma et al. (2021)	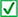	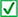	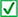	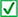	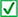	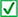	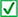	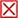	Head-mounted displays (HTC Vive)
21	Minoura et al. (2020)	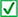	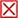	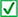	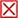	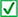	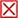	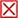	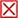	Head-Mounted Displays (Oculus Rift DK2)
22	Nataraj et al. (2020)	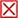	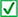	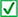	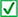	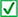	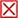	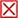	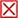	Head-mounted displays (Oculus Rift)
23	Nataraj et al. (2022)	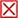	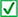	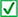	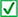	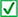	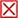	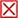	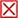	HMD Oculus Rift
24	Okumura et al. (2020)	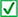	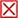	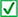	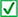	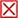	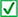	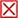	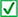	Head-mounted displays (Oculus Rift)
25	Ogawa et al. (2021)	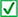	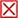	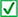	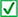	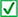	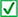	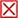	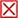	HMD Oculus Rift CV1
26	Perez-Marcos et al. (2012) - Exp1	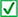	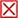	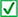	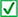	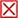	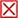	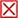	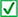	Head-mounted displays (Fakespace Wide5)
27	Perez-Marcos et al. (2012) - Exp2	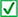	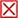	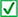	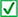	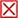	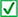	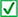	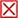	Head-mounted displays (Fakespace Wide5)
28	Perez-Marcos et al. (2012) - Exp3	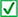	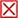	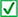	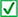	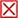	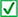	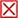	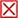	Head-mounted displays (Fakespace Wide5)
29	Petkova and Ehrsson (2008) - Exp2 & Exp3	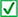	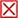	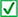	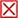	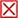	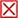	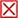	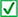	Head-Mounted Displays (Cybermind Visette Pro PAL)
30	Petkova and Ehrsson (2008) - Exp4	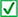	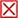	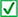	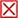	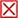	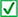	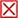	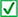	Head-mounted displays (Cybermind Visette Pro PAL)
31	Petkova et al. (2011) - Exp2	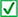	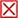	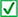	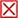	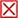	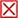	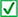	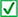	Head-mounted displays (Cybermind Visette Pro PAL)
32	Preuss and Ehrsson (2019) - Exp1	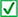	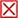	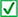	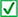	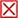	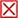	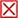	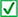	Head-mounted displays (Oculus Rift 2)
33	Preuss and Ehrsson (2019) - Exp2	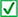	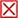	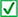	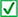	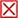	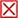	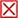	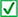	Head-mounted displays (Oculus Rift 2)
34	Preuss and Ehrsson (2019) - Exp3	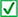	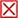	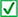	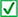	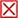	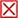	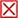	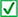	Head-mounted displays (Oculus Rift 2)
35	Pyasik et al. (2020)	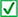	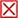	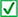	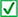	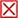	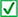	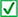	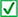	Head-mounted displays (Oculus Rift2)
36	Raz et al. (2020)	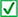	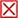	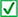	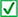	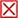	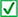	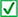	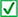	Head-mounted displays (HTC Vive)
37	Seinfeld and Müller (2020) - Exp1	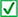	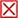	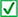	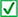	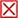	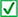	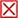	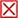	Head-mounted displays (HTC Vive)
38	Suzuki et al. (2019) - Exp1	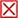	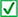	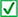	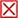	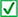	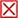	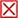	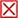	Head-mounted displays (Oculus Rift)
39	Suzuki et al. (2019) - Exp3	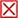	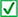	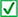	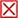	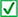	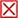	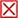	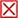	Head-mounted displays (Oculus Rift)
40	Sakurada et al. (2023)	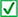	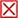	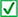	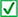	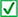	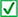	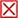	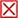	HMD HTC Vive
41	Tieri et al. (2015)	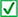	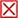	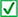	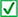	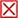	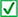	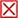	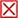	Head-mounted displays (Oculus)
42	Unruh et al. (2023)	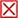	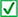	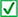	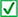	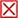	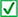	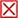	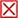	HMD HTC Vive
43	Wang et al. (2023)	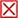	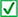	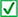	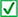	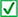	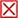	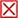	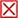	HMD HTC Vive
44	Wiesing and Zimmermann (2024) - Exp1	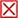	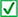	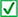	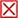	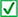	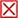	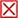	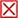	Head-mounted displays (HTC Vive Pro Eye)
45	Wiesing and Zimmermann (2024) - Exp2	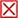	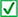	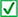	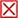	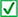	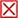	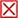	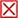	Head-mounted displays (HTC Vive Pro Eye)
46	Wiesing and Zimmermann (2024) - Exp3	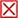	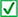	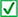	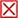	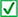	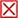	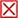	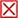	Head-mounted displays (HTC Vive Pro Eye)
47	Zhang et al. (2023)	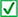	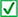	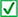	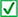	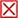	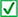	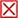	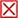	HMD HTC Vive

**FIGURE 2:**
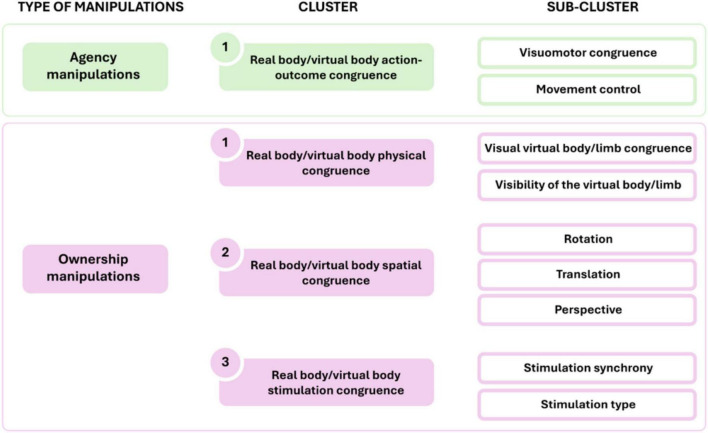
Schematic representation of virtual reality (VR) manipulations included in our review, divided into clusters and sub-clusters for the sense of agency and the sense of body ownership.

We categorized these studies based on the following criteria (see also Figure 2 and Supplementary Table 1):

(i) the type of agency manipulation employed:

• Action-outcome congruence between the movement of the real body/limb and the virtual body/limb:
  • Visuomotor congruence (e.g., the movement of the virtual body/limb and the virtual body/limb could be spatially/temporally congruent or not; Kondo et al., 2018).
  • Movement control (e.g., the virtual body/limb moved controlled by the real body/limb or not; Wiesing and Zimmermann, 2024).

(ii) the type of ownership manipulation employed:

• Physical congruence between the real body/limb and the virtual body/limb:
  • Visual virtual body/limb congruence (e.g., the virtual limb could be detached from or attached to the virtual body; Tieri et al., 2015).
  • Visibility of the virtual body/limb (e.g., a virtual limb was present or not; Okumura et al., 2020).
• Spatial congruence between the real body/limb and the virtual body/limb:
  • Rotation (e.g., the virtual limb could be rotated or not with respect to the body; Ma et al., 2021).
  • Translation (e.g., the virtual limb could be located congruently or incongruently with respect to the real body midline; Pyasik et al., 2020).
  • Perspective (e.g., the virtual body/limb could be presented in one/third-person perspective; Debarba et al., 2017).
• Congruence between the stimulation of the real body/limb and the virtual body/limb:
  • Synchrony of stimulation (e.g., the sensory stimulation to the real and virtual body could be synchronous or asynchronous; Ehrsson, 2007).
  • Type of stimulation (e.g., the sensory stimulation to the real and virtual body could be of the same or different type; Preuss and Ehrsson, 2019).

(iii) the SoA outcome measure measured:

• Explicit SoA: judgments of agency (e.g., Ma et al., 2021).
• Implicit SoA: intentional binding phenomenon (e.g., Suzuki et al., 2019).

(iv) the SoO outcome measure measured:

• Explicit SoO: judgments of ownership (e.g., Hapuarachchi et al., 2023).
• Implicit SoO: proprioceptive drift for a limb (i.e., limb-localization, e.g., Hara et al., 2015) or for the whole body (i.e., self-localization measure, e.g., Lenggenhager et al., 2007; or skin conductance; Guterstam and Ehrsson, 2012).

We first evaluated which manipulations effectively produced changes in the considered outcome measures and determined whether these modulatory effects were confined within the same dimension (e.g., agency manipulations affecting SoA measures) or extended to a generalized effect (e.g., agency manipulations influencing also SoO measures, and vice versa).

For the subset of studies reporting both explicit and implicit indexes of SoA and SoO, we also assessed the degree of agreement between these measures within each manipulation cluster (see Table 1 and Supplementary Table 1 for more details).

## 3 Results

The first section provides an overview of how implicit dimensions of SoA and SoO can be measured in virtual environments (i.e., intentional binding effects or proprioceptive drift, see below). The second section describes how agency and ownership manipulations are employed in VR studies. As mentioned, we grouped them in specific clusters.

Finally, we described the effects of these manipulations on implicit SoA and SoO measures. Since some of the included studies considered both implicit and explicit measures, we also included a section exploring how these dimensions aligned or diverged depending on the specific manipulations employed.

### 3.1 Implicit measures for evaluating SoA and SoO in VR

#### 3.1.1 Intentional binding

Sense of agency can be implicitly measured by taking advantage of the so-called *intentional binding* (IB) phenomenon. IB refers to the subjective perceived compression of the temporal interval between a self-generated action (i.e., a button press) and its external outcome (i.e., a sound). Importantly, this compression is absent or significantly reduced in the case of passive actions or action observation.

The included studies measured the IB phenomenon in VR using either the Libet clock paradigm (Haggard et al., 2002) or the temporal interval estimation task (Nolden et al., 2012). In the Libet clock paradigm, participants made temporal judgments about the timing of an action and the ensuing outcome based on a clock displayed within a virtual environment (e.g., Ma et al., 2021). Conversely, the temporal interval estimation task indexed IB by assessing participants’ perception of the temporal interval between an action and a sensory outcome presented after a variable delay: the greater the perceived temporal compression, the higher the implicit SoA (e.g., Suzuki et al., 2019; Wiesing and Zimmermann, 2024).

#### 3.1.2 Proprioceptive drift

*Proprioceptive drift* (PD) has been widely used as an implicit measure to assess SoO in both traditional and virtual environments. It refers to the phenomenon whereby the position of the real body part or the real body is perceived (i.e., self-localized) as shifted toward the location of the virtual body/body part following the embodiment procedure. This shift serves as an indirect index of the degree to which participants incorporate the virtual body or body part into their own bodily representation. In selected studies, PD was measured by comparing the real body/limb’s perceived location before and after the experimental manipulations (e.g., Pyasik et al., 2020).

#### 3.1.3 Skin conductance response

Another implicit measure of SoO can be derived from variations in physiological parameters, such as the *skin conductance response* (SCR; e.g., Tieri et al., 2015). Specifically, SCR has been employed to quantify participants’ physiological reactions to threatening or startling events (e.g., knives, Petkova and Ehrsson, 2008) directed to the virtual body or virtual limb. These responses serve as an implicit measure of SoO (depending on the functioning of a body defense system; Gallace and Bellan, 2018), with greater SCR changes indicating a stronger SoO toward the virtual body/limb (Preuss and Ehrsson, 2019).

### 3.2 Classification of agency and ownership manipulations in VR

In this section, we provide an overview of innovative VR paradigms used to selectively manipulate SoO and SoA dimensions. A schematic diagram of manipulation clusters is shown in Figure 2.

#### 3.2.1 Agency manipulations

SoA can be manipulated by varying the level of congruence between the real body/limb movements and the virtual body/limb movements. Based on the nature of these manipulations, we identified two primary clusters: *visuomotor congruence and movement control*.

##### 3.2.1.1 Visuomotor congruence

VR enables the selective manipulation of visual, proprioceptive, and tactile virtual action outcomes. This allows for the creation of scenarios where action outcomes can either align or conflict with the participants’ real movements. For example, the temporal congruence between real and virtual body movements can be manipulated by varying the *temporal congruence* (e.g., synchronicity) between the real movement and the virtual movement (Ma et al., 2021, Kondo et al., 2020a,b; Sakurada et al., 2023), by increasing or decreasing the speed pace of the virtual movement compared to the real one (Minoura et al., 2020; Nataraj et al., 2020, Nataraj et al., 2022; Wang et al., 2023). Experimental paradigms may also manipulate the *spatial congruence* between the real and the virtual movements (i.e., virtual biomechanically impossible movements, Hapuarachchi et al., 2023), or lateral shift during a reaching task (Ogawa et al., 2021) or the *congruence* of the specific movement type (i.e., a virtual body moving independently from participants’ movement, Kondo et al., 2018).

##### 3.2.1.2 Movement control

In this set of manipulations, the movements of the virtual body or limb are manipulated to either match or deviate from the participant’s motor intentions. For example, virtual hand movements can reproduce pre-recorded movements or computer-generated movements, enabling scenarios where participants observe a virtual hand performing a motor action while their own hand remains still (Suzuki et al., 2019; Wiesing and Zimmermann, 2024).

A similar manipulation was employed by Debarba et al. (2017), who compared conditions in which the avatar’s movements were actively controlled by the participant with passive observation conditions where the avatar moved independently. This approach uniquely simulated non-voluntary movements from a first-person perspective, allowing researchers to compare perceived SoA over sensory outcomes from intentional, non-voluntary, or absent actions.

#### 3.2.2 Ownership manipulations

SoO can be manipulated by varying the perceived physical appearance of the virtual body/limb. We categorized these manipulations into three clusters, each composed of different sub-clusters: *real body/virtual body physical congruence, real body/virtual body spatial congruence, and real body/virtual body stimulation congruence.*

##### 3.2.2.1 Real body/virtual body physical congruence

Virtual reality enables different manipulations of the virtual body’s appearance. *Visual virtual body/limb congruence* has been manipulated by replacing a participant’s virtual hand with non-corporeal objects, such as 3D shapes (Ma et al., 2021; Raz et al., 2020; Zhang et al., 2023, Ogawa et al., 2021), a wooden panel (Pyasik et al., 2020), or even a virtual humanoid robotic arm (Sakurada et al., 2023).

Virtual body parts (e.g., a virtual hand) can also be presented detached from the body, disrupting the continuity between the virtual and physical limb (Perez-Marcos et al., 2012; Seinfeld and Müller, 2020; Tieri et al., 2015), or elongated to implausible distances (Kilteni et al., 2012). Participants can also experience a virtual full body with distorted body parts (Kondo et al., 2020a) or corresponding to those of another individual (Guterstam and Ehrsson, 2012) or a virtual object (Heydrich et al., 2013; Lenggenhager et al., 2007).

Another manipulation implies varying the visibility of the virtual limb (*visibility of the virtual body/limb*; Okumura et al., 2020).

##### 3.2.2.2 Real body/virtual body spatial congruence

VR also allows different spatial manipulations of the position of the virtual body/limb. For instance, the virtual body/limb position may be shifted along the x-axis (i.e., *translation*; Perez-Marcos et al., 2012; Pyasik et al., 2020), *rotated* (i.e., rotation, Ma et al., 2021; Raz et al., 2020) or showed in a different *perspective* (i.e., first- or third-person perspective; Debarba et al., 2017; Petkova et al., 2011).

##### 3.2.2.3 Real body/virtual body stimulation congruence

VR enables the manipulation of the congruence of visuo-tactile, visuo-proprioceptive, or visuo-vestibular stimulations. For example, visuo-tactile stimulations can be synchronous or asynchronous on the real and virtual body/limb (*stimulation synchrony*; e.g., Guterstam and Ehrsson, 2012). Additionally, studies have explored different *stimulation types*, by comparing active versus passive self-touch in the rubber hand illusion (Hara et al., 2015).

For each cluster of agency/ownership manipulations, we described their effects on implicit SoA and SoO. In addition, we conducted an additional exploratory analysis on the alignment between implicit and self-report measurements of SoA and SoO. Specifically, for studies that included both types of measures, we assessed their degree of concordance within each manipulation cluster.

The efficacy of agency and ownership manipulations on implicit SoA and SoO are summarized in Tables 2a,b, respectively. The degree of alignment between implicit and explicit measures is reported in Tables 3a,b.

**TABLE 2a:** Efficacy of agency manipulations on implicit and explicit measures of SoA and SoO.

Dependent variable		Output measure		Efficacy agency manipulations (1) - real body/Virtual body action-outcome congruence									
Type	Number of experiments	Type	Number of measures	Total	Visuomotor congruence	Movement control							
SoA	9	Implicit measure	10	7 out of 10 (70%)	4 out of 5 (80%)	3 out of 5 (60%)							
		Explicit measure	5	3 out of 5 (60%)	3 out of 5 (60%)	N/A							
SoO	11	Implicit measure	16	5 out of 16 (31.2%)	5 out of 15 (33.3%)	0 out of 1 (0.0%)							
		Explicit measure	10	10 out of 10 (100%)	9 out of 9 (100%)	1 out of 1 (100%)							

**TABLE 2b:** Efficacy of ownership manipulations on implicit and explicit measures of SoA and SoO.

Dependent variable		Output measure		Efficacy ownership manipulations (1) - real body/virtual body physical congruence			Efficacy ownership manipulations (2) - real body/virtual body spatial congruence				Efficacy ownership manipulations (3) - real body/virtual body stimulation congruence		
Type	Number of experiments	Type	Number of measures	Total	Visual virtual body/limb congruence	Presence of the virtual body/limb	Total	Rotation	Translation	Perspective	Total	Stimulation synchrony	Stimulation type
SoA	4	Implicit measure	5	0 out of 4 (0%)	0 out of 4 (0%)	N/A	0 out of 1 (0%)	0 out of 1 (0%)	N/A	N/A	N/A	N/A	N/A
		Explicit measure	5	1 out of 4 (25%)	1 out of 4 (25%)	N/A	0 out of 1 (0%)	0 out of 1 (0%)	N/A	N/A	2 out of 2 (100%)	2 out of 2 (100%)	2 out of 2 (100%)
SoO	32	Implicit measure	47	9 out of 19 (47.4%)	8 out of 18 (44.4%)	1 out of 1 (100%)	5 out of 7 (71%)	1 out of 2 (50%)	1 out of 3 (33.3%)	2 out of 2 (100%)	14 out of 20 (70%)	10 out of 16 (62.5%)	4 out of 4 (100%)
		Explicit measure	37	14 out of 16 (87.5%)	13 out of 15 (86.7%)	1 out of 1 (100%)	2 out of 4 (50%)	1 out of 2 (50%)	1 out of 1 (100%)	0 out of 1 (0.0%)	15 out of 15 (100%)	11 out of 11 (100%)	4 out of 4 (100%)

**TABLE 3a:** Alignment between implicit and explicit SoA measures.

Total measures	Agency manipulations (1) real body/virtual body action-outcome congruence									
	Total Agreement	Visuomotor congruence agreement	Movement control agreement							
5	2 out of 5 (40%)	2 out of 5 (40%)	N/A							
**Total measures**	**Ownership manipulations (1) - real body/virtual body physical congruence**			**Ownership manipulations (2) - real body/virtual body spatial congruence**				**Ownership manipulations (3) - real body/virtual body stimulation congruence**		
	Total agreement	Visual virtual body/limb congruence agreement	Presence of the virtual body/limb agreement	Total agreement	Rotation agreement	Translation agreement	Perspective agreement	Total agreement	Stimulation synchrony agreement	Stimulation type agreement
5	3 out of 4 (75%)	3 out of 4 (75%)	N/A	1 out of 1 (100%)	1 out of 1 (100%)	N/A	N/A	N/A	N/A	N/A

**TABLE 3b:** Alignment between implicit and explicit SoO measures.

Total measures	Agency manipulations (1) Real Body/Virtual Body action-outcome congruence									
	Total Agreement	Visuomotor congruence agreement	Movement control agreement							
13	3 out of 13 (23.1%)	3 out of 12 (25%)	0 out of 1 (0%)							
**Total measures**	**Ownership manipulations (1) - Real Body/Virtual Body physical congruence**			**Ownership manipulations (2) - Real Body/Virtual Body spatial congruence**				**Ownership manipulations (3) - Real Body/Virtual Body stimulation congruence**		
	**Total Agreement**	**Visual virtual body/limb congruence agreement**	**Presence of the virtual body/limb agreement**	**Total Agreement**	**Rotation agreement**	**Translation agreement**	**Perspective agreement**	**Total Agreement**	**Stimulation synchrony agreement**	**Stimulation type agreement**
38	7 out of 16 (43.7%)	6 out of 15 (40%)	1 out of 1 (100%)	4 out of 6 (66.6%)	2 out of 2 (100%)	2 out of 3 (66.7%)	0 out of 1 (0%)	10 out of 15 (66.7%)	6 out of 11 (54.6%)	4 out of 4 (100%)

### 3.3 The impact of agency and ownership manipulations on implicit SoA

#### 3.3.1 Agency manipulations: real body/virtual body action-outcome congruence

##### 3.3.1.1 Visuomotor congruence

Four out of five experiments (80%) showed significant changes in implicit SoA following *visuomotor congruence* manipulations. Reduced congruence - whether in timing, spatial alignment, or kinematic features - generally led to reduced implicit SoA. For instance, Ma et al. (2021) found that introducing random timing between real and virtual actions significantly reduced IB. Similarly, Nataraj et al. (2020, 2022) observed a decrease in implicit SoA when the virtual limb moved more slowly than the real limb, although this effect was not replicated by Wang et al. (2023), who found no significant change. See Table 2a.

*These findings suggest that manipulating visuomotor congruence exert a strong effect on implicit SoA.*

##### 3.3.1.2 Movement control

Three out of five experiments (60%) reported significant changes in implicit SoA in response to *movement control* manipulations. Suzuki et al. (2019) revealed increased implicit SoA during voluntary and pre-recorded movements compared to the passive observation. However, these effects were not replicated by Wiesing and Zimmermann (2024), who found no significant differences across the same experimental conditions. See Table 2a.

*These findings suggest that manipulating of movement control has a moderate effect on implicit SoA.*

#### 3.3.2 Ownership manipulations: real body/virtual body physical congruence

None of the four studies manipulating physical congruence between the real and virtual body reported significant changes in implicit SoA (Ma et al., 2021; Hartfill et al., 2024; Nataraj et al., 2022; Zhang et al., 2023). See Table 2b.

*These findings suggest that physical congruence manipulations have no effect on implicit SoA.*

#### 3.3.3 Ownership manipulations: real body/virtual body spatial congruence

The single study manipulating spatial congruence between the real and virtual body (Ma et al., 2021) - involving a rotation of the virtual hand - did not report significant changes in implicit SoA. See Table 2b.

*While this may suggest that manipulating spatial alignment alone has no influence on implicit SoA, the evidence remains inconclusive due to the scarcity of studies using this manipulation.*

#### 3.3.4 Ownership manipulations: real body/virtual body stimulation congruence

Based on our review, no studies investigated the effects of real body/virtual body stimulation congruence manipulations on implicit SoA. See Table 2b.

### 3.4 The impact of agency and ownership manipulations on implicit SoO

#### 3.4.1 SoA manipulations: real body/virtual body action-outcome congruence

##### 3.4.1.1 Visuomotor congruence

Five out of fifteen experiments (33.3%) reported significant changes in implicit SoO in response to *visuomotor congruence* manipulations. Grechuta et al. (2019) observed increased implicit SoO, evidenced by greater PD toward the virtual hand and enhanced SCR, when visuomotor congruence was maintained across *spatial, temporal, and semantic* dimensions. Ogawa et al. (2021) observed increased PD toward a virtual hand in case of *spatial congruence*; no such effect was found for an abstract hand model. Sakurada et al. (2023) found that asynchronous visuomotor feedback reduced PD, but only when the virtual effector resembled a realistic hand, not when it appeared as a generic virtual object. Finally, Kondo et al. (2018) observed PD toward an avatar under movement congruence.

In contrast, a larger number of studies did not report significant effects. Subsequent experiments by Kondo et al. (2020a,b) failed to replicate their earlier findings. Ma et al. (2021) also found no PD changes when manipulating temporal congruence (e.g., congruent vs. delayed feedback), and Minoura et al. (2020) reported no SCR differences in response to variations in virtual hand speed. Similarly, Hapuarachchi et al. (2023) found no SCR effects following spatial or temporal manipulations of avatar movement. See Table 2a.

*Together, these findings indicate that visuomotor congruence manipulations can have a mild effect on implicit SoO under certain conditions, although the effects are not consistently observed across studies.*

##### 3.4.1.2 Movement control

To date, only one study has investigated the effects of movement control manipulations on implicit SoO, and it did not report significant changes. Debarba et al. (2017) compared active control versus passive observation of avatar movements and found no significant differences in SCR during threat exposure. See Table 2a.

*This suggests that manipulating movement control, on its own, may not be sufficient to modulate implicit SoO, although this conclusion remains tentative due to the limited available evidence.*

#### 3.4.2 Ownership manipulations: real body/virtual body physical congruence

Nine out of nineteen experiments (47.4%) reported significant changes in implicit SoO in response to *real body/virtual body physical congruence* manipulations. Kilteni et al. (2012) found that altering the length of the virtual arm modulated PD, with greater drift when the virtual limb appeared longer than the real one. Regarding the realism of the virtual hand, Ogawa et al. (2021) and Sakurada et al. (2023) found PD only when the virtual hand was rendered with realistic features. Similarly, Pyasik et al. (2020) reported increased PD toward a virtual hand compared to a virtual object.

In a full-body context, Lenggenhager et al. (2007) found greater body-centered PD when the illusion involved a human avatar compared to a virtual object. In out-of-body illusion paradigms, Petkova and Ehrsson (2008) reported significant SCR changes when the illusion was applied to a mannequin body compared to an object, while Guterstam and Ehrsson (2012) found significant SCR changes when the illusion involved one’s own body avatar rather than another person’s body avatar. Finally, Okumura et al. (2020) found that increasing the transparency of the virtual limb - thus enhancing visibility of the real hand - increased PD.

In contrast, several studies found no significant effects. Ma et al. (2021), Zhang et al. (2023), Raz et al. (2020) reported no PD changes when the hand was replaced by an object. Similarly, Heydrich et al. (2013) found no difference in PD between a human avatar and a virtual object.

Manipulations involving anatomical coherence also yielded mixed results: while Tieri et al. (2015) reported increased SCR following visual anatomical disconnection, this finding was not replicated in later studies (Seinfeld and Müller, 2020; Perez-Marcos et al., 2012). Kondo et al. (2020a) observed no significant changes in PD or SCR when comparing canonical and scrambled body configurations. See Table 2b.

*Together, these findings suggest that physical congruence manipulations, particularly those involving visual realism and anatomical plausibility, have a mild influence implicit SoO under specific conditions, though the overall pattern of effects remains variable across studies.*

#### 3.4.3 Ownership manipulations: real body/virtual body spatial congruence

Five out of seven experiments (71%) reported significant changes in implicit SoO following *real body/virtual body spatial congruence* manipulations. Raz et al. (2020) found that the *rotation* of the virtual hand reduced PD, while Ma et al. (2021) did not. Pyasik et al. (2020) and Frisco et al. (2024) reported that translating the virtual limb’s position modulated PD. Regarding SCR, two studies investigated the effect of perspective by comparing first-person and third-person viewpoints during virtual threat exposure (Debarba et al., 2017; Petkova et al., 2011), while Frisco et al. (2024) compared conditions in which the virtual limb was aligned or misaligned with the real limb position. Both Debarba et al. (2017), Petkova et al. (2011) found increased SCR when threats were experienced from a first-person perspective. On the other hand, Frisco et al. (2024) did not observe any difference between the two conditions. See Table 2b.

*These findings suggest that spatial congruence manipulations - particularly through limb alignment and egocentric perspective - can moderately modulate implicit SoO.*

#### 3.4.4 Ownership manipulations: real body/virtual body stimulation congruence

Fourteen out of twenty experiments (70%) showed significant effects of *real body/virtual body stimulation congruence* manipulation on implicit SoO. Most of these studies focused on the synchrony of visuotactile input. While synchronous stimulation typically increased PD toward the virtual hand (Botvinick and Cohen, 1998), some studies also reported PD in asynchronous conditions (Perez-Marcos et al., 2012; Pyasik et al., 2020; Raz et al., 2020), suggesting that asynchrony does not always abolish the illusion of ownership. However, one study reported a PD only in synchronous visuo-tactile stimulation conditions while manipulating the visibility of the virtual hand (Okumura et al., 2020).

In full-body illusion experiments, PD was consistently observed only in synchronous stimulation conditions (Heydrich et al., 2013; Lenggenhager et al., 2007). Moreover, nearly all experiments manipulating the stimulation synchrony in out-of-body illusion reported changes in SCR (Ehrsson, 2007; Petkova et al., 2011; Petkova and Ehrsson, 2008). The only exception was the study of Guterstam and Ehrsson (2012), which found no difference between synchronous and asynchronous stimulation during a body swap illusion involving another person’s body.

Stimulation type manipulations, such as active vs. passive touch, showed even higher consistency, with all four experiments reporting significant effects. For instance, Hara et al. (2015) demonstrated that PD was stronger toward the hand receiving touch during active self-touch, but not toward the hand performing the touch, indicating a role for efferent signals in ownership.

Finally, participants exposed to congruent visual-vestibular stimulation showed increased SCR to threats compared to unimodal and bimodal (visuo-tactile) incongruent stimulation, emphasizing the importance of vestibular signals in body ownership illusions (Preuss and Ehrsson, 2019). See Table 2b.

*Together, these findings suggest that stimulation manipulations moderately modulates implicit SoO, particularly in paradigms involving full-body and out-of-body illusions. Notably, asynchronous stimulation can still influence PD under certain conditions. Beyond visuotactile input, other multisensory channels, such as visuo-vestibular congruence and active self-touch, also play a significant role in shaping implicit SoO experiences.*

### 3.5 Alignment between implicit and explicit SoA across manipulation clusters

In the following section, we summarize the degree of agreement between implicit and explicit measures of SoA within each manipulation cluster.

#### 3.5.1 Agency manipulations: real body/virtual body action-outcome congruence

##### 3.5.1.1 Visuomotor congruence

Studies measuring both implicit and explicit measures of SoA in response to visuomotor congruence manipulations reveal a mild degree of accordance. (level of accordance across measures = 40%). For example, Nataraj et al. (2020; 2022) observed that manipulating the *temporal congruence* between the virtual and real actions produced changes in IB but did not affect participants’ agency ratings.

However, using a similar manipulation, Wang et al. (2023) reported the opposite results (modulation in self-report but not in implicit agency measurement). The only study reporting alignment between explicit and implicit measures of SoA was Ma et al. (2021), where temporal incongruence reduced both IB and agency ratings. See Table 3a.

*In summary, studies combining implicit and explicit measures of SoA often show inconsistencies, with some manipulations influencing explicit SoA without affecting implicit SoA, or vice versa*.

##### 3.5.1.2 Movement control

No study measured the effect of movement control manipulations on both explicit and implicit SoA in the same experimental setting.

#### 3.5.2 Ownership manipulations: real body/virtual body physical congruence

These manipulations showed a high alignment across explicit and implicit SoA measures (Ma et al., 2021; Hartfill et al., 2024; Zhang et al., 2023), with no influence on both implicit and explicit SoA in three studies out of four (level of accordance across measurements = 75%). See Table 3a.

*In summary, these studies suggest that physical congruence manipulations may not significantly modulate either explicit or implicit SoA.*

#### 3.5.3 Ownership manipulations: real body/virtual body spatial congruence

Ma et al. (2021) was the only study to include both explicit and implicit SoA measures in response to *spatial congruence* manipulations. The results aligned, showing no modulation of either explicit or implicit SoA. See Table 3a.

*In summary, the available evidence suggests that spatial congruence manipulations may not significantly modulate either explicit or implicit SoA; however, conclusions remain limited due to the inclusion of only a single study.*

#### 3.5.4 Ownership manipulations: real body/virtual body stimulation congruence

No study measured the effect of real body/virtual body stimulation congruence in the same experimental setting on explicit and implicit SoA.

### 3.6 Alignment between implicit and explicit SoO across manipulation clusters

In the following section, we summarize the degree of agreement or disagreement between implicit and explicit measures of SoO within each manipulation cluster.

#### 3.6.1 Agency manipulations: real body/virtual body action-outcome congruence

##### 3.6.1.1 Visuomotor congruence

Experiments examining the effects of *visuomotor congruence* manipulations on both explicit and implicit SoO showed limited accordance (accordance level across measurements = 25%). In Grechuta et al.’s (2019) study, the manipulation of *temporal, spatial, and semantic congruence* modulated both explicit and implicit SoO (PD and SCR). Similarly, Sakurada et al. (2023) showed an agreement between PD and SoO ratings.

Conversely, Ma et al. (2021), Hapuarachchi et al. (2023), Ogawa et al. (2021), Kondo et al. (2020a,b) reported inconsistency between implicit and explicit SoO measurements under *temporal, spatial or movement congruence* manipulation, with no modulation in implicit SoO, while explicit SoO were affected. See Table 3b.

*Altogether, these studies suggest that while visuomotor manipulations may modulate explicit ownership ratings, its effects on implicit SoO measures remain limited.*

##### 3.6.1.2 Movement control

Only one study (Debarba et al., 2017) assessed the effect of movement control manipulations on both explicit and implicit SoO within the same experimental setting, reporting no agreement between the two measures. See Table 3b.

*While this suggests a potential misalignment between implicit and explicit outcomes, broader conclusions are limited by the availability of a single study.*

#### 3.6.2 Ownership manipulations: real body/virtual body physical congruence

Experiments investigating the impact of physical congruence between the real and virtual body on both implicit and explicit SoO measures revealed mild accordance (according level across measurements = 43.7%).

Several studies that manipulated *visual congruence* between the virtual body or limb and the participant’s real body reported that explicit SoO ratings were sensitive to these manipulations, whereas PD and SCR remained largely unaffected (Kondo et al., 2020a; Perez-Marcos et al., 2012; Seinfeld and Müller, 2020; Heydrich et al., 2013; Ma et al., 2021; Zhang et al., 2023), while Ogawa et al. (2021) found the opposite effect (changes in PD, while explicit ownership ratings remained similar).

However, other studies found convergence between explicit and implicit measures. For instance, Pyasik et al. (2020), Sakurada et al. (2023), Raz et al. (2020) observed alignment between PD and explicit ownership ratings in response to visual congruence manipulations. Tieri et al. (2015) reported that both SCR and explicit ownership ratings were sensitive to manipulations involving body disconnection, but such an effect was not replicated in Seinfeld and Müller’s (2020).

Lenggenhager et al. (2007) demonstrated alignment between implicit and explicit SoO measures, showing that both responded to physical congruence manipulations. However, despite employing a similar paradigm, this alignment was not replicated by Heydrich et al. (2013).

Finally, manipulating the visibility of the virtual hand produced similar changes in implicit and explicit SoO measurements (Okumura et al., 2020). See Table 3b.

*Taken together, although some inconsistencies remain, the observed mild agreement between implicit and explicit SoO measures suggests a mild level of alignment in response to physical congruence manipulations.*

#### 3.6.3 Ownership manipulations: real body/virtual body spatial congruence

With respect to the spatial body congruence cluster, the according level across measures was moderate (accordance level across measurements = 66.7%). *Rotation* manipulations affect implicit and explicit SoO measurement (although with different direction across studies; Ma et al., 2021, Raz et al., 2020). *Translation* manipulations affected both implicit and explicit SoO in Pyasik et al. (2020), but not in Frisco et al. (2024). Finally, *perspective* manipulations modulated implicit but not explicit SoO measures in Debarba et al. (2017). See Table 3b.

*Overall, these findings suggest a moderate level of alignment between implicit and explicit SoO measures in response to spatial congruence manipulations, although the direction and consistency of effects can vary depending on the specific type of manipulation employed.*

#### 3.6.4 Ownership manipulations: real body/virtual body stimulation congruence

Experiments manipulating *stimulation congruence* on both explicit and implicit SoO measures showed a moderate agreement (accordance level across measurements = 66.7%). *Stimulation type* manipulations consistently influenced both explicit and implicit SoO measures (Hara et al., 2015; Preuss and Ehrsson, 2019). However, *stimulation synchrony* applied to specific body parts yielded more conflicting results, with visuo-tactile synchronicity modulating explicit SoO but not implicit SoO (Perez-Marcos et al., 2012; Hara et al., 2015; Pyasik et al., 2020; Raz et al., 2020; Heydrich et al., 2013).

In contrast, studies focusing on full-body or out-of-body illusions found partial evidence of concordance, with PD aligning with explicit ownership ratings depending on the synchronicity of stimulation in Lenggenhager et al. (2007), Ehrsson (2007), but not in Heydrich et al. (2013) with modulation present only at the explicit level.

*These findings indicate that stimulation congruence manipulations produce a moderate alignment between changes in implicit and explicit SoO measures, though the degree of concordance varies depending on the specificity of the stimulation (e.g., localized vs. full-body).*

## 4 Discussion

Virtual reality has revolutionized the study of the SoA and SoO by creating immersive and highly customizable paradigms characterized by experimental manipulations extending well beyond traditional laboratory settings’ limitations. In this review, we summarized how these manipulations may affect SoA and SoO measures, focusing on their effects on implicit indexes and their alignment with explicit ratings.

### 4.1 Advancing the study of SoA and SoO through innovative VR paradigms

Agency manipulations were categorized into two sub-clusters: visuomotor congruence and movement control. In movement control manipulations, observing movements from a first-person perspective while dissociating them from voluntary control sheds light on how sensory feedback influences the SoA in the absence of volitional input (Suzuki et al., 2019; Wiesing and Zimmermann, 2024). The visuomotor congruence cluster included several manipulations, such as temporal congruence (e.g., movement synchronicity, Ma et al., 2021), spatial congruence (e.g., Hapuarachchi et al., 2023), and semantic congruence (Grechuta et al., 2019). Ownership manipulations range from basic visual (Tieri et al., 2015) and spatial (Debarba et al., 2017) changes to finely tuned multisensory congruence alterations, in terms of visuo-tactile or visuo-vestibular stimulation (Petkova and Ehrsson, 2008; Preuss and Ehrsson, 2019). Notably, all the studies leverage VR to create bodily illusions that are impossible to re-create in non-virtual settings. Outstanding examples include virtual bodies or limbs performing implausible movements (Hapuarachchi et al., 2023), the embodiment of an “invisible avatar” through visuomotor synchronization (Kondo et al., 2018, 2020a,2020b), and avatars featuring elongated virtual limbs that extend far beyond normal human anatomical limits (Kilteni et al., 2012). There has also been considerable interest in the possibility of remapping control of a virtual body part using movements from a different body region with a similar joint angle—for example, controlling a virtual left arm using the motion of the right thumb.

Finally, VR allows for manipulation of visual perspective, enabling control of a virtual body from a third-person viewpoint. This has prompted investigations into how third-person embodiment compares to the canonical body ownership experience from a first-person perspective (Debarba et al., 2017). Furthermore, in traditional laboratory settings, multisensory stimulation—particularly synchronous visuo-tactile input—is the gold standard for inducing ownership, as exemplified by the rubber hand illusion (RHI). However, this passive paradigm limits the exploration of motor contributions to SoO. While active versions of the RHI exist, they are constrained in scope. In contrast, VR easily facilitates the induction of ownership through visuomotor synchrony, thus broadening the range of ownership manipulations to include both passive and active conditions. Finally, studies combining agency and ownership manipulations and collecting various SoA and SoO measures provide new insights into their interrelation. That is, VR offers a unique advantage, as it enables flexible and targeted manipulations of ownership (e.g., the appearance of the virtual body) and agency (e.g., visuomotor synchronicity)- related features. This allows researchers to explore how changes in ownership affect agency, and how alterations in agency, in turn, shape ownership. Such approaches not only enhance experimental control but also support more ecologically valid investigations into the complex relationship between SoA and SoO.

### 4.2 Modulation of implicit SoA and SoO: manipulation clusters showed mixed results

Our review examined whether and how the manipulation clusters described in the previous paragraph modulate the implicit dimensions of SoA and SoO.

We found evidence that agency manipulations exerted different levels of influence on implicit SoA, with visuomotor congruence showing large effects and movement control showing moderate effects. On the other hand, ownership manipulations had no significant effect. For example, substituting a virtual hand with an object did not affect the magnitude of the IB effect: participants can experience SoA even over non-corporeal objects if the visuomotor congruence between the real and the virtual movement is preserved (Ma et al., 2021; Unruh et al., 2023). These results suggest that the physical congruence of the virtual body/limb (compared to the real body/limb) is not critical for the implicit agency experience. Instead, implicit SoA appears to rely more on low-level sensorimotor cues, such as visuomotor synchrony.

Results from implicit SoO measurements were mixed. Agency manipulations generally had mild impact on these measures. Prior research has examined the interplay between agency and ownership by comparing active (participant-controlled) versus passive (experimenter-controlled) movements, and synchronous versus asynchronous visuomotor feedback (Kalckert and Ehrsson, 2012, 2014). For instance, in case of active RHI, asynchronous movement between the real and fake hand tends to disrupt PD toward the fake hand. This discrepancy may stem from differences in how ownership is induced and measured across paradigms. Illusions in VR using visuomotor synchrony may be weaker than those in the active RHI, which involves a physically present fake hand. Full-body visuomotor incongruence, typically assessed via self-localization, was generally less effective than part-based measures like proprioceptive drift (Grechuta et al., 2019; Kondo et al., 2020a,b). Similarly, implicit measures such as skin conductance responses to virtual threats often showed null effects, possibly due to insufficient illusion strength (Hapuarachchi et al., 2023; Kondo et al., 2020a,b). Overall, unlike the more robust findings from the active RHI, the link between visuomotor congruence and implicit SoO in VR remains inconclusive and requires further study.

Similarly, ownership manipulations yielded often heterogeneity in modulating implicit SoO. Regarding manipulations of physical congruence between the real and virtual body, approximately half of the studies that altered visual congruence of the virtual limb or body reported significant changes in implicit SoO. However, findings were inconsistent even when using comparable experimental paradigms. For example, presenting a virtual hand detached from the body modulated SCR in one study (Tieri et al., 2015) but not in another (Seinfeld and Müller, 2020). On the other hand, real body/virtual body spatial and stimulation congruence manipulations showed greater effectiveness. Manipulating perspective, such as viewing a virtual avatar from a third-person perspective, diminished SoO, as reflected in reduced physiological threat responses (Debarba et al., 2017). Similarly, synchronous stimulation was a critical factor for increasing ownership toward a virtual avatar in full-body and out-of-body illusions, evident through self-localization drift and SCR measures (Lenggenhager et al., 2007).

Notably, stimulation congruence often resulted in null modulatory effects on the PD, that emerged toward the virtual hand, even during asynchronous stimulation conditions (Pyasik et al., 2020; Hara et al., 2015; Raz et al., 2020). These findings support the hypothesis that merely observing a virtual limb from a first-person perspective, aligned with the real body, is sufficient to perceive the limb as one’s own, regardless of the congruence of visuo-tactile stimulation, challenging traditional rubber hand illusion results (Makin et al., 2008). A possible explanation for these results lies in the experimental settings of virtual rubber hand illusion compared to the classic one. In the virtual version of the illusion, participants view only the virtual hand from an egocentric and immersive perspective. This setup may enhance the reliability of visual-proprioceptive information, which alone is sufficient to induce PD toward the virtual hand, even in the absence of synchronous tactile stimulation (Perez-Marcos et al., 2012; Raz et al., 2020). Additional evidence supporting this explanation comes from studies where limb alignment was manipulated. In these experiments, spatial translation of the virtual hand reduces implicit SoO (PD), regardless of the synchronicity of stimulation (Pyasik et al., 2020). These findings support the possibility of establishing a hierarchy of manipulations influencing SoO in VR environments. Among these, spatial congruence manipulations emerged as more impactful than stimulation congruence (suggesting an important role of higher-order spatial representation systems in SoO; Moseley et al., 2012). However, this hierarchy primarily applies to manipulations involving single body parts (e.g., the hand or limb) and to PD measures. In contrast, stimulation congruence remains critical for inducing full-body illusions, and SCR was selectively modulated based on stimulation modality (synchronous vs. asynchronous; Ehrsson, 2007; Lenggenhager et al., 2007; Petkova et al., 2011; Petkova and Ehrsson, 2008).

### 4.3 Comparing implicit and explicit measures of SoA and SoO for each manipulation cluster

To provide a comprehensive understanding of how innovative VR-based agency and ownership manipulations influence SoA and SoO, we also examined the alignment between implicit and explicit measures—typically assessed through self-report questionnaires administered during or after the experimental experience—when both types of measures were available. Although implicit and explicit measures of SoA and SoO are not entirely independent, they are conceptually and empirically distinct (Moore et al., 2012 Abdulkarim and Ehrsson, 2016). Notably, studies that include both types of measures frequently report a lack of correlation and dissociation in outcomes, and the degree of alignment between them remains a topic of debate (Dewey and Knoblich, 2014; Tosi et al., 2023). As part of our review, we conducted a descriptive comparison to examine alignment between implicit and explicit measures across studies. Overall, consistency between the two was low. Explicit measures were generally more sensitive to experimental manipulations, while implicit measures often showed non-significant effects—especially across different manipulation clusters in SoA and SoO studies in VR.

Although comparing implicit and explicit measures was not a primary aim of this review, several studies in our sample reported both types of outcomes, allowing for exploratory insights into their relationship. Within this limited subset, alignment between implicit and explicit SoA measures ratings was relatively mild (accordance level = 40%) and often inconsistent, even within the same manipulation clusters. Some studies reported changes in IB but not in agency ratings (Nataraj et al., 2020, 2022), and vice versa (Unruh et al., 2023). In contrast, ownership-related manipulations - especially those altering physical or spatial congruence - consistently showed no effect on SoA, with both implicit and explicit measures aligned in showing null results. Notably, in the movement control cluster, no studies assessed both implicit and explicit SoA within the same experimental context - limiting our ability to evaluate potential alignment or dissociation in this domain.

A general dissociation was also found between implicit and explicit SoO measures, particularly when visuomotor (accordance = 23.1%) or physical body (accordance = 43.7%) congruence was manipulated. In these cases, effects appeared mainly in explicit measures, with implicit responses largely unaffected. This pattern was especially evident under asynchronous stimulation involving only parts of the virtual body, where explicit ownership ratings changed, but implicit measures did not (Perez-Marcos et al., 2012; Raz et al., 2020). However, during full-body illusions with synchronous stimulation, implicit and explicit measures were more closely aligned, highlighting the importance of visuo-tactile synchrony for inducing a strong ownership illusion (e.g., Ehrsson, 2007; Lenggenhager et al., 2007).

These findings further suggest that implicit and explicit measures capture distinct underlying mechanisms, likely reflecting differences between low-level and high-level cognitive processes—an idea extensively discussed in the literature on SoA (Synofzik et al., 2013) and SoO (Longo, 2015). Self-report ratings tend to reflect high-level, conscious evaluations of SoA and SoO, making them particularly sensitive to bodily incongruities introduced by VR paradigms. Importantly, a key distinction characterizes paradigms implemented in virtual reality versus those in traditional laboratory settings. Although VR is designed to simulate real-world conditions, the experience of interacting with a virtual body is inherently novel and unfamiliar. This novelty, stemming from the atypical experience of embodying a virtual avatar, may affect low-level mechanisms that typically underlie readaptation of SoA and SoO, typically captured by implicit measurements. For instance, in the case of SoA, explicit measures are often driven by judgments of agency—postdictive evaluations shaped by beliefs, expectations, and contextual cues—rather than the more immediate, sensorimotor-based feeling of agency (Synofzik et al., 2008, 2013). Because VR represents a simulated and often unfamiliar experience, low-level predictive mechanisms may not fully activate or adapt, which could explain the relative insensitivity of implicit measures to agency manipulations in these settings. In contrast, post-experience evaluations—relying on postdictive processes—may be more readily available for explicit judgment. This distinction may help explain why explicit measures more consistently capture effects of agency manipulations in VR settings.

Despite this consideration, we observed a considerable variability in how implicit and explicit measurements respond differently to ownership and agency manipulations, even within the same cluster. Therefore, the precise mechanisms underlying the differences between implicit and explicit components of SoA and SoO remain unclear, and further research specifically addressing these distinctions is needed, particularly with the added advantages of new experimental VR paradigms.

### 4.4 Decoupling agency and ownership: experimental insights from VR manipulations

Although preliminary due to the limited number of studies incorporating both implicit and explicit measurements and the heterogeneity of applied manipulations, our review highlights compelling questions about the relationship between agency and ownership.

Our findings demonstrate that although SoA and SoO are inherently related, they can be independently modulated at the implicit level depending on the type of virtual manipulation employed (Braun et al., 2018). This dissociation is particularly evident in studies examining how ownership manipulations affect implicit agency. For instance, replacing a participant’s virtual hand with an incongruent object but preserving real-time voluntary movement leaves implicit SoA measures unchanged (Ma et al., 2021). Such findings support the so-called “indepndence model” of SoA and SoO, which posits that SoA and SoO rely on distinct underlying processes (Tsakiris et al., 2010). Notably, these findings also support the idea that SoA can persist even when the SoO is reduced or absent. For example, participants may still report a SoA over a virtual hand placed in an anatomically incongruent position, as long as its movements remain synchronous with their own actions—suggesting that SoA does not necessarily depend on SoO and can be maintained over representations no longer integrated as part of the body (Kalckert and Ehrsson, 2012).

However, agency manipulations, such as visuomotor incongruence, mildly affect implicit SoO - indicating that such cross-dimensional modulation is limited but not absent. These findings may offer support for both the additive view—where SoA encompasses SoO—and interactive models between SoA and SoO. The additive model posits that voluntary movement enhances body perception, thereby strengthening the SoO. The interactive model, instead, propose a bidirectional relationship between the two constructs. Supporting this view, a recent fMRI meta-analysis investigating brain regions involved in SoO and SoA found that, while some areas selectively respond to one dimension, others—such as the left middle insula—exhibit shared activation across both, suggesting interdependency across the two dimensions (Seghezzi et al., 2019a). In this context, our review highlights that a virtual body unresponsive to one’s actions may undermine implicit SoO suggesting that motor-related (efferent) signals contributing to agency also facilitate a more coherent and embodied ownership experience.

The review presents several limitations that should be acknowledged when interpreting the current findings and their implications for models of agency and ownership. First, this review focused exclusively on studies that included implicit measures of SoA and/or SoO, occasionally alongside explicit measures, but did not consider studies based solely on explicit assessments. While this allowed for a deeper examination of pre-reflective bodily processes, it may have excluded relevant insights from purely explicit paradigms. Second, relatively few studies combined both agency and ownership manipulations or included SoA and SoO measures within the same experimental design, limiting our ability to draw strong conclusions about their dynamic interaction. Third, many studies lacked detailed reporting or control of key experimental factors known to influence the emergence of agency—such as prior familiarization with the avatar, the degree of embodiment, or baseline levels of sensorimotor congruency—potentially introducing variability that could affect the consistency of observed effects.

Additionally, future studies could also include additional implicit measures, such as temperature modulation (previously used in body ownership manipulation; e.g., Kammers et al., 2011; Moseley et al., 2008, but which led to conflictual results in non-immersive investigation; e.g., de Haan et al., 2017), or histamine reactivity (Barnsley et al., 2011), to expand the range of implicit assessments.

## 5 Conclusion

Virtual reality has advanced the study of SoA and SoO mechanisms through novel experimental paradigms and illusions involving a wide range of manipulations between real and virtual body experiences. However, the efficacy of these manipulations in implicit measurements remains variable, with some clusters proving more effective than others. While explicit measures of SoA and SoO generally show sensitivity to these manipulations, implicit and explicit results often conflict, raising questions about whether they represent different aspects of these two dimensions. Further research is needed to resolve the discrepancies between implicit and explicit measurements and to clarify the conditions under which these manipulations influence both types of measures. Pinpointing the manipulations that specifically influence agency or ownership will offer valuable insights for future research aiming to uncover the mechanisms underlying SoO and SoA.

## Data Availability

The original contributions presented in this study are included in this article/Supplementary material, further inquiries can be directed to the corresponding authors.

## Footnotes

1 VR is a computer-generated environment that simulates senses like sight, sound, and touch, immersing users in realistic or imaginative worlds, enabling interaction and navigation as if physically present (Lanier, 2017).

2
https://www.rayyan.ai/

## References

[B1] Abdulkarim Z. Ehrsson H. H. (2016). No causal link between changes in hand position sense and feeling of limb ownership in the rubber hand illusion. *Attent. Percept. Psychophys.* 78 707–720. 10.3758/s13414-015-1016-0 26555651 PMC4744264

[B2] Antoš D. Švec T. Hořínková J. Bartečková E. (2024). Borders of physical self in virtual reality: A systematic review of virtual hand position discrepancy detection. *Front Psychiatry* 15:1455495. 10.3389/FPSYT.2024.1455495 39834571 PMC11743482

[B3] Armel K. C. Ramachandran V. S. (2003). Projecting sensations to external objects: Evidence from skin conductance response. *Proc. R. Soc B* 270 1499–1506. 10.1098/rspb.2003.2364 12965016 PMC1691405

[B4] Barnsley N. McAuley J. H. Mohan R. Dey A. Thomas P. Moseley G. L. (2011). The rubber hand illusion increases histamine reactivity in the real arm. *Curr. Biol.* 21 R945–R946. 10.1016/j.cub.2011.10.039 22153159

[B5] Blanke O. Slater M. Serino A. (2015). Behavioral, neural, and computational principles of bodily self-consciousness. *Neuron* 88 145–166. 10.1016/j.neuron.2015.09.029 26447578

[B6] Botvinick M. Cohen J. (1998). Rubber hands ‘feel’ touch that eyes see. *Nature* 391 756–756. 10.1038/35784 9486643

[B7] Braun N. Debener S. Spychala N. Bongartz E. Sörös P. Müller H. H. O. (2018). The senses of agency and ownership: A review. *Front. Psychol.* 9:535. 10.3389/fpsyg.2018.00535 29713301 PMC5911504

[B8] Caspar E. A. Cleeremans A. Haggard P. (2015). The relationship between human agency and embodiment. *Conscious. Cogn.* 33 226–236. 10.1016/j.concog.2015.01.007 25655206

[B9] de Haan A. M. Van Stralen H. E. Smit M. Keizer A. Van der Stigchel S. Dijkerman H. C. (2017). No consistent cooling of the real hand in the rubber hand illusion. *Acta Psychol.* 179 68–77. 10.1016/j.actpsy.2017.07.003 28735225

[B10] De Vignemont F. (2011). Embodiment, ownership and disownership. *Conscious. Cogn.* 20 82–93. 10.1016/j.concog.2010.09.004 20943417

[B11] Debarba H. G. Bovet S. Salomon R. Blanke O. Herbelin B. Boulic R. (2017). Characterizing first and third person viewpoints and their alternation for embodied interaction in virtual reality. *PLoS One* 12:e0190109. 10.1371/journal.pone.0190109 29281736 PMC5744958

[B12] Dewey J. A. Knoblich G. (2014). Do implicit and explicit measures of the sense of agency measure the same thing? *PLoS One* 9:110118. 10.1371/journal.pone.0110118 25330184 PMC4199671

[B13] Ehrsson H. H. (2007). The experimental induction of out-of-body experiences. *Science* 317:1048. 10.1126/science.1142175 17717177

[B14] Farrer C. Valentin G. Hupé J. M. (2013). The time windows of the sense of agency. *Conscious. Cogn.* 22 1431–1441. 10.1016/j.concog.2013.09.010 24161792

[B15] Frisco F. Bruno V. Romano D. Tosi G. (2024). I am where I believe my body is: The interplay between body spatial prediction and body ownership. *PLoS One* 19:e0314271. 10.1371/journal.pone.0314271 39666650 PMC11637335

[B16] Gallace A. Bellan V. (2018). The parietal cortex and pain perception: A body protection system. *Handb. Clin. Neurol.* 151 103–117. 10.1016/B978-0-444-63622-5.00005-X 29519454

[B17] Gallace A. Spence C. (2014). *In Touch with the Future.* Oxford: Oxford University Press. 10.1093/acprof:oso/9780199644469.001.0001

[B18] Gallace A. Ngo M. K. Sulaitis J. Spence C. (2011). “Multisensory presence in virtual reality: Possibilities & limitations,” in *Multiple Sensorial Media Advances and Applications: New Developments in MulSeMedia*, eds Ghinea G. Gulliver S. Andres F. (Hershey, PA: Information Science Reference), 1–38. 10.4018/978-1-60960-821-7.ch001

[B19] Gallagher S. (2000). Philosophical conceptions of the self: Implications for cognitive science. *Trends Cognit. Sci.* 4 14–21. 10.1016/S1364-6613(99)01417-5 10637618

[B20] Girondini M. Montanaro M. Gallace A. (2024a). Spatial tactile localization depends on sensorimotor binding: Preliminary evidence from virtual reality. *Front. Hum. Neurosci.* 18:1354633. 10.3389/fnhum.2024.1354633 38445099 PMC10912179

[B21] Girondini M. Montanaro M. Lega C. Gallace A. (2024b). Spatial sensorimotor mismatch between the motor command and somatosensory feedback decreases motor cortical excitability. A transcranial magnetic stimulation-virtual reality study. *Eur. J. Neurosci.* 60 5348–5361. 10.1111/ejn.16481 39171623

[B22] Grechuta K. Ulysse L. Rubio Ballester B. Verschure P. F. M. J. (2019). Self beyond the body: Action-driven and task-relevant purely distal cues modulate performance and body ownership. *Front. Hum. Neurosci.* 13:412150. 10.3389/fnhum.2019.00091 30949038 PMC6435571

[B23] Guterstam A. Ehrsson H. H. (2012). Disowning one’s seen real body during an out-of-body illusion. *Consciousn. Cogn.* 21 1037–1042. 10.1016/j.concog.2012.01.018 22377139

[B24] Haggard P. (2017). Sense of agency in the human brain. *Nat. Rev. Neurosci.* 18 197–208. 10.1038/nrn.2017.14 28251993

[B25] Haggard P. Clark S. Kalogeras J. (2002). Voluntary action and conscious awareness. *Nat. Neurosci.* 5 382–385. 10.1038/nn827 11896397

[B26] Hapuarachchi H. Ishimoto H. Kitazaki M. Sugimoto M. Inami M. (2023). Temporal visuomotor synchrony induces embodiment towards an avatar with biomechanically impossible arm movements. *I-Perception* 14:20416695231211699. 10.1177/20416695231211699 37969571 PMC10631331

[B27] Hara M. Pozeg P. Rognini G. Higuchi T. Fukuhara K. Yamamoto A. (2015). Voluntary self-touch increases body ownership. *Front. Psychol.* 6:1509. 10.3389/fpsyg.2015.01509 26617534 PMC4621401

[B28] Hartfill J. Bormann F. Riebandt K. Kühn S. Steinicke F. (2024). Objective agency measurement of different hand appearances in virtual reality with intentional binding. *Virtual Real.* 29:14. 10.1007/s10055-024-01085-x

[B29] Heydrich L. Dodds T. J. Aspell J. E. Herbelin B. Bülthoff H. H. Mohler B. J. (2013). Visual capture and the experience of having two bodies - evidence from two different virtual reality techniques. *Front. Psychol.* 4:66408. 10.3389/fpsyg.2013.00946 24385970 PMC3866547

[B30] Ho J. T. Preller K. H. Lenggenhager B. (2020). Neuropharmacological modulation of the aberrant bodily self through psychedelics. *Neurosci. Biobehav. Rev.* 108 526–541. 10.1016/j.neubiorev.2019.12.006 31816361

[B31] Holle H. McLatchie N. Maurer S. Ward J. (2011). Proprioceptive drift without illusions of ownership for rotated hands in the “rubber hand illusion” paradigm. *Cogn. Neurosci*. 2, 171–178. 10.1080/17588928.2011.603828 24168532

[B32] Kalckert A. Ehrsson H. H. (2014). The moving rubber hand illusion revisited: Comparing movements and visuotactile stimulation to induce illusory ownership. *Consciousn. Cogn.* 26 117–132. 10.1016/j.concog.2014.02.003 24705182

[B33] Kalckert A. Ehrsson H. (2012). Moving a rubber hand that feels like your own: A dissociation of ownership and agency. *Front. Hum. Neurosci.* 6:19533. 10.3389/fnhum.2012.00040 22435056 PMC3303087

[B34] Kammers M. P. M. Rose K. Haggard P. (2011). Feeling numb: Temperature, but not thermal pain, modulates feeling of body ownership. *Neuropsychologia* 49 1316–1321. 10.1016/j.neuropsychologia.2011.02.039 21354190

[B35] Kammers M. P. M. Verhagen L. Dijkerman H. C. Hogendoorn H. De Vignemont F. Schutter D. J. L. G. (2009). Is this hand for real? Attenuation of the rubber hand illusion by transcranial magnetic stimulation over the inferior parietal lobule. *J. Cognit. Neurosci.* 21 1311–1320. 10.1162/jocn.2009.21095 18752397

[B36] Kilteni K. Groten R. Slater M. (2013). The Sense of embodiment in virtual reality. *Presence Teleoperators Virtual Environ.* 22 373–387. 10.1162/PRES_a_00124

[B37] Kilteni K. Maselli A. Kording K. P. Slater M. (2015). Over my fake body: Body ownership illusions for studying the multisensory basis of own-body perception. *Front. Hum. Neurosci.* 9:141. 10.3389/FNHUM.2015.00141 25852524 PMC4371812

[B38] Kilteni K. Normand J. M. Sanchez-Vives M. V. Slater M. (2012). Extending body space in immersive virtual reality: A very long arm illusion. *PLoS One* 7:e40867. 10.1371/journal.pone.0040867 22829891 PMC3400672

[B39] Kokkinara E. Slater M. López-Moliner J. (2015). The effects of visuomotor calibration to the perceived space and body, through embodiment in immersive virtual reality. *ACM Transact. Appl. Percept.* 13 1–22. 10.1145/2818998

[B40] Kondo R. Sugimoto M. Minamizawa K. Hoshi T. Inami M. Kitazaki M. (2018). Illusory body ownership of an invisible body interpolated between virtual hands and feet via visual-motor synchronicity. *Sci. Rep.* 8 1–8. 10.1038/s41598-018-25951-2 29765152 PMC5954161

[B41] Kondo R. Tani Y. Sugimoto M. Inami M. Kitazaki M. (2020a). Scrambled body differentiates body part ownership from the full body illusion. *Sci. Rep.* 10 1–11. 10.1038/s41598-020-62121-9 32210268 PMC7093408

[B42] Kondo R. Tani Y. Sugimoto M. Minamizawa K. Inami M. Kitazaki M. (2020b). Re-association of Body Parts: Illusory ownership of a virtual arm associated with the contralateral real finger by visuo-motor synchrony. *Front. Robot. AI* 7:26. 10.3389/frobt.2020.00026 33501195 PMC7805900

[B43] Lanier J. (2017). *Dawn of the new everything: A journey through virtual reality*. Random House.

[B44] Lenggenhager B. Tadi T. Metzinger T. Blanke O. (2007). Video ergo sum: Manipulating bodily self-consciousness. *Science* 317 1096–1099. 10.1126/science.1143439 17717189

[B45] Longo M. R. (2015). Implicit and explicit body representations. *Eur. Psychol.* 20 6–15. 10.1027/1016-9040/a000198

[B46] Ma K. Qu J. Yang L. Zhao W. Hommel B. (2021). Explicit and implicit measures of body ownership and agency: Affected by the same manipulations and yet independent. *Exp. Brain Res.* 239 2159–2170. 10.1007/s00221-021-06125-5 33974114

[B47] Makin T. R. Holmes N. P. Ehrsson H. H. (2008). On the other hand: Dummy hands and peripersonal space. *Behav. Brain Res.* 191 1–10. 10.1016/j.bbr.2008.02.041 18423906

[B48] Matamala-Gomez M. Donegan T. Bottiroli S. Sandrini G. Sanchez-Vives M. V. Tassorelli C. (2019). Immersive virtual reality and virtual embodiment for pain relief. *Front. Hum. Neurosci.* 13:279. 10.3389/fnhum.2019.00279 31551731 PMC6736618

[B49] Minoura M. Kojima K. Nomura S. Nishiyama Y. Kawai T. Gunji Y. P. (2020). Virtual hand with ambiguous movement between the self and other origin: Sense of ownership and “other-produced” agency. *J. Visual. Exp.* 10.3791/61755 33191926

[B50] Moore J. W. (2016). What is the sense of agency and why does it matter? *Front. Psychol.* 7:1272. 10.3389/fpsyg.2016.01272 27621713 PMC5002400

[B51] Moore J. W. Fletcher P. C. (2012). Sense of agency in health and disease: A review of cue integration approaches. *Conscious. Cogn.* 21 59–68. 10.1016/j.concog.2011.08.010 21920777 PMC3315009

[B52] Moore J. W. Middleton D. Haggard P. Fletcher P. C. (2012). Exploring implicit and explicit aspects of sense of agency. *Conscious. Cogn.* 21 1748–1753. 10.1016/j.concog.2012.10.005 23143153 PMC3566545

[B53] Moseley G. L. Gallace A. Spence C. (2012). Bodily illusions in health and disease: Physiological and clinical perspectives and the concept of a cortical ‘body matrix’. *Neurosci. Biobehav. Rev.* 36 34–46. 10.1016/j.neubiorev.2011.03.013 21477616

[B54] Moseley G. L. Olthof N. Venema A. Don S. Wijers M. Gallace A. (2008). Psychologically induced cooling of a specific body part caused by the illusory ownership of an artificial counterpart. *Proc. Natl. Acad. Sci. U. S. A.* 105 13169–13173. 10.1073/pnas.0803768105 18725630 PMC2529116

[B55] Mottelson A. Muresan A. Hornbæk K. Makransky G. (2023). A systematic review and meta-analysis of the effectiveness of body ownership illusions in virtual reality. *ACM Trans. Comput. Hum. Interact.* 30 1–42. 10.1145/3590767

[B56] Nataraj R. Sanford S. Liu M. Harel N. Y. (2022). Hand dominance in the performance and perceptions of virtual reach control. *Acta Psychol.* 223 2626–2636. 10.1016/j.actpsy.2022.103494 35045355 PMC11056909

[B57] Nataraj R. Sanford S. Shah A. Liu M. (2020). Agency and performance of reach-to-grasp with modified control of a virtual hand: Implications for rehabilitation. *Front. Hum. Neurosci.* 14:126. 10.3389/fnhum.2020.00126 32390812 PMC7191072

[B58] Nolden S. Haering C. Kiesel A. (2012). Assessing intentional binding with the method of constant stimuli. *Conscious. Cogn.* 21 1176–1185. 10.1016/j.concog.2012.05.003 22726692

[B59] Ogawa N. Narumi T. Hirose M. (2021). Effect of avatar appearance on detection thresholds for remapped hand movements. *IEEE Trans. Visual. Comput. Graph.* 27 3182–3197. 10.1109/TVCG.2020.2964758 31940540

[B60] Okumura K. Ora H. Miyake Y. (2020). Investigating the hand ownership illusion with two views merged in. *Front. Robot AI* 7:49. 10.3389/frobt.2020.00049 33501217 PMC7805733

[B61] Pavani F. Spence C. Driver J. (2000). Visual capture of touch: Out-of-the-body experiences with rubber gloves. *Psychol. Sci.* 11 353–359. 10.1111/1467-9280.00270 11228904

[B62] Perez-Marcos D. Sanchez-Vives M. V. Slater M. (2012). Is my hand connected to my body? The impact of body continuity and arm alignment on the virtual hand illusion. *Cogn. Neurodyn.* 6 295–305. 10.1007/s11571-011-9178-5 24995046 PMC4079845

[B63] Petkova V. I. Ehrsson H. H. (2008). If i were you: Perceptual illusion of body swapping. *PLoS One* 3:e3832. 10.1371/JOURNAL.PONE.0003832 19050755 PMC2585011

[B64] Petkova V. I. Khoshnevis M. Ehrsson H. H. (2011). The perspective matters! Multisensory integration in egocentric reference frames determines full-body ownership. *Frontiers in Psychology* 2:8981. 10.3389/fpsyg.2011.00035 21687436 PMC3108400

[B65] Preuss N. Ehrsson H. H. (2019). Full-body ownership illusion elicited by visuo-vestibular integration. *J. Exp. Psychol.* 45 209–223. 10.1037/xhp0000597 30589357

[B66] Pyasik M. Ciorli T. Pia L. (2022). Full body illusion and cognition: A systematic review of the literature. *Neurosci. Biobehav. Rev.* 143:104926. 10.1016/J.NEUBIOREV.2022.104926 36341941

[B67] Pyasik M. Tieri G. Pia L. (2020). Visual appearance of the virtual hand affects embodiment in the virtual hand illusion. *Sci. Rep.* 10:5412. 10.1038/s41598-020-62394-0 32214171 PMC7096421

[B68] Raz G. Gurevitch G. Vaknin T. Aazamy A. Gefen I. Grunstein S. (2020). Electroencephalographic evidence for the involvement of mirror-neuron and error-monitoring related processes in virtual body ownership. *NeuroImage* 207:116351. 10.1016/j.neuroimage.2019.116351 31733375

[B69] Rognini G. Blanke O. (2016). Cognetics: Robotic interfaces for the conscious mind. *Trends Cognit. Sci.* 20 162–164. 10.1016/j.tics.2015.12.002 26750512

[B70] Saito N. Takahata K. Murai T. Takahashi H. (2015). Discrepancy between explicit judgement of agency and implicit feeling of agency: Implications for sense of agency and its disorders. *Conscious. Cognit.* 37 1–7. 10.1016/j.concog.2015.07.011 26253893

[B71] Sakurada K. Kondo R. Nakamura F. Kitazaki M. Sugimoto M. (2023). Investigating the perceptual attribution of a virtual robotic limb synchronizing with hand and foot simultaneously. *Frontiers in Virtual Real.* 4:1210303. 10.3389/frvir.2023.1210303

[B72] Sanchez-Vives M. V. Spanlang B. Frisoli A. Bergamasco M. Slater M. (2010). Virtual hand illusion induced by visuomotor correlations. *PLoS One* 5:e10381. 10.1371/journal.pone.0010381 20454463 PMC2861624

[B73] Seghezzi S. Giannini G. Zapparoli L. (2019a). Neurofunctional correlates of body-ownership and sense of agency: A meta-analytical account of self-consciousness. *Cortex* 121 169–178. 10.1016/j.cortex.2019.08.018 31629195

[B74] Seghezzi S. Zirone E. Paulesu E. Zapparoli L. (2019b). The brain in (Willed) action: A meta-analytical comparison of imaging studies on motor intentionality and sense of agency. *Front. Psychol.* 10:804. 10.3389/fpsyg.2019.00804 31031676 PMC6473038

[B75] Seinfeld S. Müller J. (2020). Impact of visuomotor feedback on the embodiment of virtual hands detached from the body. *Sci. Rep.* 10:22427. 10.1038/s41598-020-79255-5 33380732 PMC7773737

[B76] Suzuki K. Lush P. Seth A. K. Roseboom W. (2019). Intentional binding without intentional action. *Psychol. Sci.* 30 842–853. 10.1177/0956797619842191 31023161

[B77] Synofzik M. Vosgerau G. Newen A. (2008). I move, therefore I am: A new theoretical framework to investigate agency and ownership. *Conscious. Cogn.* 17 411–424. 10.1016/j.concog.2008.03.008 18411059

[B78] Synofzik M. Vosgerau G. Voss M. (2013). The experience of agency: An interplay between prediction and postdiction. *Front. Psychol.* 4:127. 10.3389/fpsyg.2013.00127 23508565 PMC3597983

[B79] Tieri G. Tidoni E. Pavone E. F. Aglioti S. M. (2015). Body visual discontinuity affects feeling of ownership and skin conductance responses. *Sci. Rep.* 5:17139. 10.1038/srep17139 26602036 PMC4658534

[B80] Tosi G. Mentesana B. Romano D. (2023). The correlation between proprioceptive drift and subjective embodiment during the rubber hand illusion: A meta-analytic approach. *Q. J. Exp. Psychol.* 76 2197–2207. 10.1177/17470218231156849 36880657

[B81] Tsakiris M. (2010). My body in the brain: A neurocognitive model of body-ownership. *Neuropsychologia* 48 703–712. 10.1016/j.neuropsychologia.2009.09.034 19819247

[B82] Tsakiris M. Longo M. R. Haggard P. (2010). Having a body versus moving your body: Neural signatures of agency and body-ownership. *Neuropsychologia* 48 2740–2749. 10.1016/j.neuropsychologia.2010.05.021 20510255

[B83] Tsakiris M. Prabhu G. Haggard P. (2006). Having a body versus moving your body: How agency structures body-ownership. *Conscious. Cogn.* 15 423–432. 10.1016/j.concog.2005.09.004 16343947

[B84] Turk D. J. Van Bussel K. Waiter G. D. Macrae C. N. (2011). Mine and me: Exploring the neural basis of object ownership. *J. Cognit. Neurosci.* 23 3657–3668. 10.1162/jocn_a_00042 21557652

[B85] Unruh F. Vogel D. Landeck M. Lugrin J.-L. Latoschik M. E. (2023). Body and time: Virtual embodiment and its effect on time perception. *IEEE Trans. Visual. Comp. Graph*. 29, 2626–2636. 10.1109/TVCG.2023.3247040 37027744

[B86] Wang L. Huang M. Yang R. Qin C. Han J. Liang H. N. (2023). Effect of reaching movement modulation on experience of control in virtual reality. *Int. J. Hum. Comput. Interact.* 40 6740–6757. 10.1080/10447318.2023.2290382

[B87] Wiesing M. Zimmermann E. (2024). Intentional binding – Is it just causal binding? A replication study of Suzuki et al. (2019). *Consciousn. Cogn.* 119:103665. 10.1016/j.concog.2024.103665 38354485

[B88] Wold A. Limanowski J. Walter H. Blankenburg F. (2014). Proprioceptive drift in the rubber hand illusion is intensified following 1 HzTMS of the left EBA. *Front. Hum. Neurosci.* 8:87143. 10.3389/FNHUM.2014.00390/BIBTEX PMC4045244 24926247

[B89] Zhang J. Huang M. Yang R. Wang Y. Tang X. Han J. (2023). Understanding the effects of hand design on embodiment in virtual reality. *Artif. Intell. Eng. Design Anal. Manufact.* 37:e10. 10.1017/S0890060423000045

